# Motivating antibiotic stewardship in Bangladesh: identifying audiences and target behaviours using the behaviour change wheel

**DOI:** 10.1186/s12889-021-10973-9

**Published:** 2021-05-22

**Authors:** Leanne E. Unicomb, Fosiul Alam Nizame, Mohammad Rofi Uddin, Papreen Nahar, Patricia J. Lucas, Nirnita Khisa, S. M. Salim Akter, Mohammad Aminul Islam, Mahbubur Rahman, Emily K. Rousham

**Affiliations:** 1 International Centre for Diarrhoeal Disease Research, Bangladesh (icddr,b), Dhaka, Bangladesh; 2 Department of Global Health and Infection, Brighton and Sussex Medical School, Sussex University, Brighton, UK; 3 School for Policy Studies, University of Bristol, Bristol, UK; 4 Paul G. Allen School for Global Health, Washington State University, Pullman, WA USA; 5 Centre for Global Health and Human Development, School of Sport, Exercise and Health Sciences, Loughborough University, Loughborough, UK

**Keywords:** Intervention, Antibiotic stewardship, Bangladesh, Antibiotic resistance

## Abstract

**Background:**

South Asia is a hotspot for antimicrobial resistance due largely to over-the-counter antibiotic sales for humans and animals and from a lack of policy compliance among healthcare providers. Additionally, there is high population density and high infectious disease burden. This paper describes the development of social and behavioural change communication (SBCC) to increase the appropriate use of antibiotics.

**Methods:**

We used formative research to explore contextual drivers of antibiotic sales, purchase, consumption/use and promotion among four groups: 1) households, 2) drug shop staff, 3) registered physicians and 4) pharmaceutical companies/medical sales representatives. We used formative research findings and an intervention design workshop with stakeholders to select target behaviours, prioritise audiences and develop SBCC messages, in consultation with a creative agency, and through pilots and feedback. The behaviour change wheel was used to summarise findings.

**Results:**

Workshop participants identified behaviours considered amenable to change for all four groups. Household members and drug shop staff were prioritised as target audiences, both of which could be reached at drug shops. Among household members, there were two behaviours to change: suboptimal health seeking and ceasing antibiotic courses early. Thus, SBCC target behaviours included: seek registered physician consultations; ask whether the medicine provided is an antibiotic; ask for instructions on use and timing. Among drug shop staff, important antibiotic dispensing practices needed to change. SBCC target behaviours included: asking customers for prescriptions, referring them to registered physicians and increasing customer awareness by instructing that they were receiving antibiotics to take as a full course.

**Conclusions:**

We prioritised drug shops for intervention delivery to all drug shop staff and their customers to improve antibiotic stewardship. Knowledge deficits among these groups were notable and considered amenable to change using a SBCC intervention addressing improved health seeking behaviours, improved health literacy on antibiotic use, and provision of information on policy governing shops. Further intervention refinement should consider using participatory methods and address the impact on profit and livelihoods for drug shop staff for optimal compliance.

## Background

South and South East Asia are hotspots for antimicrobial resistance (AMR) [1] and newly evolved AMR organisms from other regions have spread rapidly across the world [2]. Contributing factors include availability of cheap, locally manufactured over-the-counter antibiotics for humans and animals, a lack of compliance with standards among healthcare providers, weak regulatory system, and high population density [3] (WHO, 2015). While per capita antibiotic consumption rates in low- and middle-income countries (LMICs) have been lower than those in high income countries, these are increasing dramatically in line with increases in gross domestic product [4]. Human antibiotic consumption rates are predicted to increase globally by 200% from 2015 to 2030 [5]. In South Asia, dispensing antibiotics without a prescription is common [6–8]. Community members and healthcare providers’ understanding of antibiotics and their mode of action is limited [9, 10].

In Bangladesh, community members access antibiotics for themselves and their animals through a healthcare system that is pluralistic and less institution-based than elsewhere [11, 12]. Unqualified healthcare providers are a major source of health care for the poor and disadvantaged [13, 14]. These providers can include drug shop staff that have no recognised qualification. It is estimated that Bangladesh has 200,000 retail drug shops and approximately 50% of those are unlicensed [15]. While little is known about quantities of antibiotics dispensed through the various channels in the healthcare system, a recent study reported that 29% of the antibiotic prescriptions came from qualified doctors and 63% from unqualified healthcare providers [16]. Recent studies conducted by our group suggest that there is a considerable volume of antibiotics dispensed without a prescription, often by unqualified providers [17]. Antibiotics administered by these groups are shorter courses and not appropriate for the illness [18], which can contribute to AMR.

From the supply-side, previous studies on antibiotic dispensing in Bangladesh have reported polypharmacy, detected among 25% of prescriptions from rural hospital outpatient clinic doctors [16]. Overprescribing, using unnecessarily expensive drugs and dispensing drugs without a prescription is common [15, 19]. Additionally, drug sellers and healthcare providers are exposed to aggressive marketing strategies, especially through pharmaceutical company representatives [20]. From the demand side, limited data are available on household-level antibiotic use for humans and their animals/livestock [16, 21].

Bangladesh has taken steps to address poor compliance with accepted standards for antibiotic prescription with the launch of the Bangladesh Pharmacy Model Initiative (BPMI) for all medication categories in 2016, which also forms a key part of the national action plan on AMR. It includes standards for drug outlet personnel, premises, dispensing, storage, hygiene, record keeping, disposal and allowable products. To date there have been 193 model pharmacies and 154 model medicine shops developed [22, 23]. The BPMI requires retail outlets to provide medications only to customers with a prescription, dispensed by staff with pharmacy qualifications and training [24]. The National Drug Policy, 2016 [25] provides the legal requirements for drug dispensing: it “prohibits sales and distribution of drugs without prescription from registered physician to ensure rational use of drugs” and “Retail sales of drugs is prohibited without prescription by registered physicians/ veterinarians other than the over-the-counter drugs”.

Antibiotic stewardship programmes, to optimise antibiotic dispensing and consumption, have predominantly been implemented in high income countries and in hospitals [26]. A review of programmes/interventions to reduce antibiotic prescribing in LMICs reported that the majority (*n* = 36) took place in hospitals and 9 in pharmacies, with mixed success [27]. In the review, the authors noted that few stewardship interventions included more than one healthcare provider group or setting [27]. In Tanzania, a programme to improve antibiotic stewardship targeted drug shops as part of accreditation in as many as 9000 premises which included training staff in appropriate antibiotic dispensing [28]. Ten years after the programme began, audit studies have detected residual needless antibiotic dispensing among one-third of the shops [29].

Understanding the behaviours, service and economic priorities of healthcare providers, and the needs of consumers is therefore central to developing an effective strategy to engage in antimicrobial stewardship to reduce antibiotic resistance [17, 30]. This study is part of a larger project that aimed to inform government policy and identify pathways to behaviour change among groups from the antibiotic supply and demand sides [31]. The specific objective of this paper is to describe the development of social and behavioural change communication (SBCC) messages aimed to increase the appropriate use of antibiotics. We explored antibiotic sales, purchase, use/consumption and promotion to identify contextual drivers among four groups: 1) households, 2) drug shop staff, 3) registered physicians and 4) pharmaceutical companies/medical representatives. We used formative study findings from this research in conjunction with outcomes from an intervention design workshop with stakeholder to identify target behaviours, and in consultation with a creative agency, to prioritise audiences and refine SBCC messages.

## Methods

In this study we collected data for integration into the first four steps of the behaviour change wheel, used to formulate behaviour change interventions [32, 33]. These steps include 1) defining the problem in behavioural terms, identifying who performs the behaviours and listing all other behaviours that might influence the problem behaviour; 2) selecting the target behaviour, 3) specifying the target behaviour, and 4) identifying what needs to change [33].

Formative data collection provided information for step 1 [17, 30] on current key behaviours. Following the formative research, steps 2 to 4 were undertaken through an intervention design workshop and the co-creation of intervention resources with a creative agency.

### Formative study

The objectives of the formative study were to a) determine the drivers of household decision making on healthcare consultations and antibiotic purchase and consumption; b) determine the practices among registered and unqualified healthcare providers (doctors, drug shops); and examine the interactions of drug shops and doctors with pharmaceutical company representatives. We selected one rural community in Tangail district and one urban community in Gazipur district. The sites were selected where households had access to a range of drug shops and health care facilities.

Formative respondent selection, data collection and data analysis details have been described previously [17, 31]. In brief, between May 2017 to January 2018 data were collected through face-to-face and in-depth interviews with groups that were identified as key actors in community-level antibiotic dispensing, use, consumption and promotion based on review of existing literature. The key actors for data collection comprised household members (*n* = 48); drug shop staff who were either unqualified (*n* = 13) or had up to 12 months training (*n* = 14); registered physicians in human and veterinary medicine (*n* = 12); auxiliary healthcare providers (*n* = 7) and key informants including pharmaceutical company representatives and non-government organisations (e.g., Bangladesh Druggist and Chemist Association) (*n* = 13). Respondents were selected to comprise as diverse a group as possible and thus provide a wide range of responses, based on qualitative research principles. Interviews were audio-recorded and transcribed in Bangla. A third of all transcripts were translated in full into English by native Bangla-speaking researchers. The remaining Bangla transcripts were coded in Bangla, and then translated into English and shared with the research team.

### Intervention design workshop

The objective of the workshop was to obtain stakeholder expert input in the selection of key behaviours and audiences for an intervention. In conjunction with a local creative agency, Visual Communications Ltd. (VISCOM, https://www.viscombd.com), we invited 60 people to participate in a 1 day workshop in February, 2018 including stakeholders to potentially cover all that likely have a stake in reducing AMR such as from the Government Directorate General of Health Services, Directorate General of Drug Administration and the Department of Livestock Services; pharmaceutical industry and medical representatives; the Bangladesh Chemist and Druggist Association, local non-governmental organisations (NGOs), drug shop owners, implementing partners, and the research team; 33 attended.

We shared emerging findings from the formative studies with the workshop participants relating to healthcare seeking behaviours and antibiotic supply, dispensing and consumption. Based on research findings and input from the workshop participants the study team, in association with the design agency, discussed different specific content, target audiences and behaviour options. Workshop attendees participated in group work focusing on each of the four possible target audiences (householders, drug shop staff, registered physicians, pharmaceutical companies/medical representatives) to a) define the problem in behavioural terms (who performs behaviours, other behaviours that might influence the problem behaviour); b) select target behaviour, specifying target behaviour, identify what needs to change and c) propose SBCC messages. Each group had a facilitator from the research team and a rapporteur who recorded discussions on template Powerpoint slides which were presented to workshop participants. Suggestions provided during the group work feedback session were recorded. The workshop concluded with a voting exercise where each attendee was provided with stickers to vote for their first and second priority among the four groups/ target audiences.

### Working with a creative agency to develop key messages in collaboration with the research team

VISCOM collaborated with the research team to collate the suggested target behaviours we wished to change and supporting messages from the workshops. Draft intervention resources were designed in English and Bangla and the research team provided feedback. Further feedback was obtained through a display session in the Environmental Interventions Unit at the International Centre for Diarrhoeal Disease Research, Bangladesh (icddr,b) to solicit suggestions from experienced researchers who have worked on a wide range of SBCC interventions.

After incorporating initial feedback, intervention resources were piloted in two convenience sample selected drug shops each from one rural and one urban setting (total 4) in July 2018. Interviews with one drug shop staff and one customer per drug shop were used to explore understanding of messages and anticipated impact of the intervention resources on behaviour. This feedback was used to revise intervention materials. Revised intervention resources were subsequently distributed to 95 convenience sample selected drug shops in September 2018 from the same two geographic areas and remained for a month. From the 95 pharmacies, 50 were randomly selected and interviews conducted with one drug shop staff and one customer from each drug shop 1 month after intervention resource delivery. Interviews followed an open-ended questionnaire similar to that used in the earlier pilot among 4 shops, collected demographic information, and sought suggested further changes on appearance or content.

### Data analysis

**Fig. 1:**
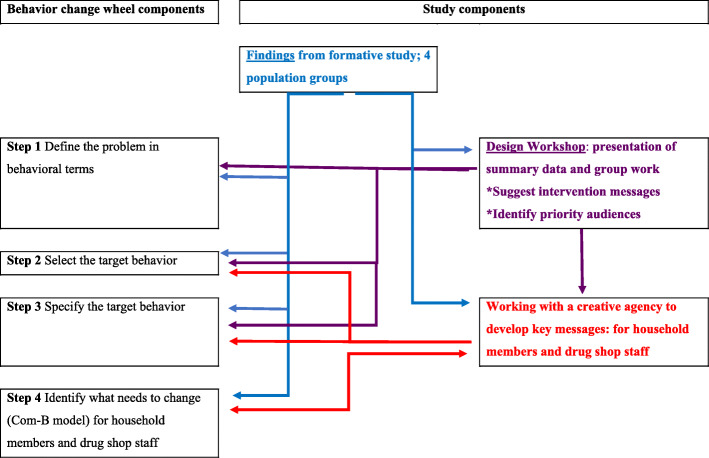
Schematic of study components

Data collected from formative studies were analysed using framework analysis, as described previously [17]. In addition to guiding data collection, data were mapped using the behaviour change wheel [32] for steps 1 to 4, as described by Munir et al. [33]; these are mapped against study components in Fig. 1. For steps 1–3, data analysed from formative studies and data collected during the workshop were summarised (Table 1). For step 4, for the prioritised audiences, we used the Capability, Opportunities, Motivation-Behaviour (Com-B) framework [32] to summarise data to provide detail on the behaviours that need to change (Table 2).

**Table 1:** Behaviour change and intervention components for the four groups from the intervention design workshop

Behaviours		Proposed intervention messages**
Current practice/contextual drivers*	Selected behaviours to change	
***Households***		
• Don’t know what an antibiotic is • Don’t know what an antibiotic is for • Drug shops common first point for health advice • Purchase drug without prescription/ without doctor’s advice; sometimes ask for antibiotics by name • Underage purchaser and proxy for patient • Use peer group prescribed antibiotic • Antibiotics are stopped when symptoms disappear or if patients consider that they don’t work • Don’t reuse drugs for subsequent illnesses • Have high level knowledge of medicine expiry	• Consult registered physicians or health workers • Ask the healthcare provider if they have been given antibiotics and why • Buy full course of antibiotic and continue according to the prescription • Ask about dose frequency and duration/instruction on use • Choose a pharmacy that maintains quality drugs	• Consult with registered physician for prescription; (who are they and how do patients know if doctor is registered) • Buy full course of drugs and complete the course; Follow your prescription to recover • If you don’t follow instructions, it will cost you more ultimately • If you suffer from any adverse effects, consult your doctor immediately • Buy medicines from reputable company
***Drug shops***		
• Most common source of medical advice; minor illness • Dispensing without government license • Unqualified staff dispensing antibiotics, with short (6 weeks) or no training • Dispense antibiotic without seeing the patient/animal, prescribe over phone • Follow elite doctor prescriptions when asked for advice • Do not always give a name to the type of illness • Often advise and sell drugs for livestock • Some drug sellers know antibiotic generation no. • Few drug sellers are familiar with antibiotic resistance” • No clear idea on consumer rights	• Ask for prescription before dispensing antibiotics • Refer customers to registered physicians • Increase awareness among patients; tell purchasers which is an antibiotic- stress importance of taking full course • Recruit qualified staff • Do not change the medicine when prescribed by a registered physician	• Stop selling antibiotic without registered doctor’s prescription • Tell customer that you are providing an antibiotic • Dispense full antibiotic course and give instructions for consumption • Unnecessary antibiotic use is harmful • Check expiry date before dispensing drugs
***Registered physicians***		
• See patients with more severe disease • Give little time during consultation • Prescribe over telephone • Overprescribe antibiotics and provide unnecessary tests, to make profit • Prescribe to keep patients happy • Prescribe poor quality antibiotics • Follow prescriptions of senior/renowned doctors • Limited knowledge of antibiotic generations • Don’t request tests before prescribing antibiotics/prescribe for viral infections • Don’t counsel on antibiotic use or the consequences of overuse • Not up to date on current literature • Take incentives from pharmaceutical companies • Motivated by medical representatives to prescribe antibiotics	• Do not provide antibiotic prescription when not necessary; consult recent recommendation information • Resist prescribing later generation antibiotics • Reinforce that the full course is important to complete • Prescribe only when the patient presents at the consultation	• Follow knowledge and practice on up to date information on antibiotic resistance • Do not provide antibiotic prescription when not necessary • Provide instructions for consumption, include the need for a full course
***Pharmaceutical companies/medical representatives***		
• Have regional offices and numerous staff for product promotion and distribution • Are aware of government policy • Have thorough understanding of antibiotic resistance • Purported provision of incentives to doctors to prescribe, drug shop staff to sell • Range of product quality • Representatives have monthly visit quotas for doctors to promote products, distribute sample medicines to physicians • Provide products to drug shops sometimes on credit, some with incentives (e.g. one free box) • Review prescriptions for marketing strategy	**Companies** • Modify business strategy • Deliver quality training • Package antibiotics according to course • Include warning messages on packet **Medical representatives** • Motivate the drug seller to sell full courses of antibiotic • Don’t review prescriptions at doctors’ offices, drug shops • Don’t motivate patients to purchase drugs in front of doctor’s offices, drug shops	• Promote full courses of antibiotic for better health • Ensure the proper use of antibiotics • Ensure profit, protect yourself and others • Do quality business for community health

*data from formative studies; **data from workshop working groups

**Table 2:** Using the Com-B model to inform households/customer and drug shop staff intervention to improve antibiotic stewardship

Behaviour to change	Capability	Opportunity	Motivation
***Households***			
**Suboptimal health seeking** • Purchase drugs without prescription; sometimes ask for antibiotics by name since unqualified healthcare providers first point for health • Use peer group prescribed antibiotic	**Psychological** • Limited knowledge of difference between registered and unqualified physicians • Self-prescription • Accessibility, cost, symptom severity drive health seeking behaviour **Physical** • Easy access to (free) health advice from drug shop staff	**Physical** • Need information on who and where are registered physicians • Registered physician/population is low **Social** • Males are decision makers on expenditure, visit drug shop • Social norm to visit drug shop first	**Reflective** • Want quality healthcare for the family at reasonable cost • Want adequate information **Automatic** • Sometimes question advice
**Antibiotics are stopped** • When symptoms disappear • When patients consider that they don’t work	**Psychological** • Don’t know what an antibiotic is/ its use • Limited understanding of how antibiotics work **Physical** • Full course not purchased	**Physical** • Need information about the importance of why drug is prescribed • Need information on dosage and timing, need for full course • Cost barrier to full course **Social** • Social norm to stop medications when disease is ‘cured’	**Reflective** • Empower to ask about treatment, cost **Automatic** • Trust drug shop staff
***Drug shop staff***			
**Antibiotic dispensing** • Without government license^a^ • Without prescription from registered physician^b^ • Without seeing the patient/animal, prescribe over phone • By unqualified staff/ with short (6 weeks) or no training^a^ • Follow elite doctor prescriptions when asked for advice • Do not always give dosing instructions^a^ • Not familiar with antibiotic resistance	**Psychological** • Limited knowledge of the policies, rules and penalties • Need information on policy for prescribing and minimum staff qualification	**Physical** • Educate drug sellers on antibiotic resistance • Address financial implications on their businesses • Intervention can replace medical representatives as a source of trusted, unbiased information on antibiotics • Policy is specific about staff qualification	**Reflective** • Respected in the community **Automatic** • Dispense multiple times during longer illnesses

^a^as outlined in the Bangladesh Model Pharmacy initiative [24]; ^b^as outlined in the Bangladesh National Drug Policy, 2016

## Results

### Defining the problem in behavioural terms among four groups from formative studies (step 1)

Contextual drivers of antibiotic use among household members, and the inappropriate prescribing and dispensing practices of healthcare professionals and drug shop staff have been published [17, 30]. Among household members, an important contextual driver was that most did not what know an antibiotic was or what antibiotics were for. Household members and healthcare providers reported that antibiotics were stopped when symptoms disappeared. Antibiotics were often purchased by proxies for ill household members/patients. Underage children and adolescents were also able to purchase antibiotics from drug shops. Drug shop staff, regardless of training and qualification, in addition to dispensing antibiotics prescribed by registered physicians, dispensed antibiotics as over-the-counter medications. Counter to common assumptions, households did not report storing or re-using old antibiotics.

A contextual driver of antibiotic dispensing among drug shop staff was that they regularly sold prescription drugs including antibiotics without a government license, in conflict with government policy. Drug shop staff advised and dispensed drugs to patients and their proxies, who were usually family members. Unqualified drug shop staff reported that they followed antibiotic prescribing patterns of registered physicians, referred to as ‘elite’ doctors/‘*boro* (big)’ doctors.

Patients were more likely to consult registered physicians for more severe diseases, or after an initial treatment had failed. Travel costs and distance to health facilities or clinics were barriers to seeking earlier consultation with qualified professionals [17]. However, doctors were reported to give little time during consultation, prescribe drugs including antibiotics over the telephone and patients perceived that the additional costs of consulting a doctor or undergoing recommended tests were to enhance profits and were often viewed as unnecessary [30].

Pharmaceutical companies were reported to provide incentives to doctors for prescribing their company’s antibiotics. In contrast with registered doctors and drug shop staff, pharmaceutical representatives were fully aware of the BPMI policy and had a thorough understanding of antibiotic resistance.

### Selecting and specifying target key behaviours (steps 2 and 3)

Based on data from formative studies on contextual drivers, participants in the intervention design workshop selected and specified behaviours that were amenable to change (Table 1).

*Household members****:*** should be encouraged to consult registered physicians and be pro-active in obtaining information about dispensed medications such as whether they are antibiotics. For those receiving antibiotics, the dose, frequency and duration of the course should be explained. Patients/consumers should be encouraged to take a full course of antibiotics obtained from a drug shop that sells quality medicines.

*Drug shop staff****:*** should be encouraged to ask the customer for a prescription before dispensing antibiotics; referring them to doctors when they do not provide a prescription. Drug shop staff were considered to have a responsibility to increase awareness among patients, particularly on the importance of taking a full course of antibiotics. Stakeholders thought that there should be incentives for drug shops to recruit qualified staff.

*Registered physicians****:*** should be encouraged to base practices on current recommendations to reduce unnecessary prescribing, especially avoiding prescribing multiple and higher generation antibiotics than is necessary. Doctors were considered as important information sources that should reinforce the importance of completing a full course of antibiotics.

*Pharmaceutical companies/medical representatives****:*** Stakeholders acknowledged the potential for companies and their representatives to maintain a viable business whilst playing a role in antibiotic stewardship. Suggestions included: modifications to the business strategy of companies; delivering quality training to all representatives and designing antibiotic packaging in a way that would encourage sale of a full course.

### Identifying priority audiences

Among 28 participants who voted for first and second priority audiences to target, 19 voted for household members, 12 of which considered this population as their number one priority and 16 voted for drug shop staff, 8 of which voted for this group as their first priority. Doctors were the third priority target audience with 13 votes and pharmaceutical companies/representatives were the lowest priority with 8 votes. During the post-workshop discussions, the research team and creative agency members concluded that using drug shops as a venue for intervention delivery had potential to address both the first and second priority audiences, thereby providing an opportunity to maximise intervention impact.

### Capability, opportunity and motivation to improve antibiotic stewardship among priority audiences (com-B model, step 4)

In line with government policy and guidelines [25, 34], to improve antibiotic stewardship, the target behaviors were to sell and purchase fewer antibiotics and to sell and consume antibiotics as full courses only.

Among households, there were two main behaviours that needed to change: suboptimal health seeking and early cessation of antibiotic treatment. When assessing *capabilities*, a recurring theme was knowledge. Most household members could not distinguish a qualified from an unqualified provider and made decisions on who to visit primarily on disease severity considerations. They also had limited knowledge about antibiotics and their mode of action. Thus, *opportunities* exist for developing an SBCC that strengthens knowledge that can empower household members with potential to impact responsible antibiotic consumption. These include encouraging household members to ask about the medicines that they receive, and ask about timing, dosage, and course duration. *Motivation* for this group can be encouraging them to seek appropriate healthcare advice and medicines by appealing to potential financial burden and accessibility to registered physicians.

When identifying drug shop staff behaviours that needed to change, there were similar *capability* issues evident as limited knowledge of the government policy including the BPMI on licensing, staff qualification and adequate provision of information on antibiotics to customers. They lacked knowledge about antibiotic resistance. Some of these knowledge gaps can be filled using an educational SBCC. Drug shop staff are likely to remain the first line of access to health care in many communities, presenting an *opportunity* to have them serve as an information source on antibiotic use/dose/timing for customers. For an educational campaign that is located at drug shops, it must *motivate* drug shop staff by addressing financial concerns of potential lost business by encouraging household members to seek care from registered physicians and by acknowledging their status in the community. While the BPMI is clear about staff qualifications, there does not appear to be evidence of enforcement, which could act as a motivator (Table 2).

### Intervention resource development and pilots for household members and drug shop staff

The National Drug Policy, 2016 states ‘To prohibit sales and distribution of drugs without prescription from *registered physician* to ensure rational use of drugs”. During the pilot phase of intervention resource development, there was considerable discussion on how to convey ‘registered physician’. Household members often referred to their local drug shop staff as a doctor or ‘small doctor’ [17]. Village doctor is also a common term used for a rural health practitioner, a post that is not considered a registered physician. To overcome existing ambiguity, we decided to pilot ‘(Bachelor of Medicine, Bachelor of Surgery) MBBS doctor’ for the intervention resources and measure understanding of this term among drug shop staff and customers.

Among the messages suggested during the intervention design workshop, the research team and VISCOM team prioritised those that we thought would resonate with the two selected audiences. For drug shop staff, these were related to asking for prescriptions, referral to registered physicians, increase in client/customer awareness when they were receiving an antibiotic, and the need for a full course.

#### Pilot feedback

Pilot drug shop staff respondents were all male, between 20 and 62 years of age and education status ranged from Secondary School Certificate (approximately grade 10) to master’s degree, with most having a Higher School Certificate (approximately grade 12) qualification. All 50 respondents received and displayed the intervention materials and found them simple, clear and easy to read. Among the recommendations on antibiotics, 5 thought they would be difficult, 5 thought they would need time to integrate recommendations and the remainder thought the recommended behaviours were reasonable. There were suggestions on format (color, number of messages and font size).

Among the customers, 40 were male, 10 were female, ranging in age from 20 to 65 years. Some had no education, and most had attended between grade 5 and Higher School Certificate with one completing a master’s degree. The vast majority (45) found that the messages were simple and easy to understand and were able to repeat the key messages. However, they found recommendations were different from their usual practices reporting that they buy antibiotics directly as it is easier and saves money and they didn’t see a justification for visiting a registered physician for simple diseases. The majority (40) reported that medicines were recommended to them by the drug shop staff. Similar to drug shop staff materials, customers recommended some format revisions such as using more color, including fewer messages and using a larger font.

**Fig. 2:**
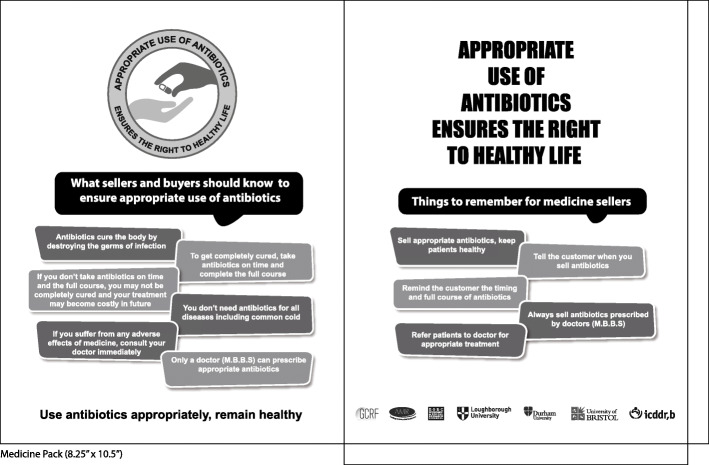
Example intervention resource: Medicine pack*. * produced as a paper bag for customers to carry dispensed antibiotics and other medicines, provided by drug retail shops – to raise awareness of when customers were receiving an antibiotic

Revised intervention resources included the following messages (example in Fig. 2), for drug shop staff: tell the customer when you sell them antibiotics, remind them of timing and completing the full course, always sell antibiotics prescribed by a doctor (MBBS), refer patients to doctors for the appropriate treatment, tell customers to report side effects to doctors. For customers messages included: antibiotics cure illness by killing germs, take a full course and follow dose and timing to be cured, not taking a full course may cause your disease to return and cost more money, antibiotics are not needed for all diseases, only a MBBS doctor can prescribe antibiotics.

## Discussion

Among the four groups examined, each contributed to poor antibiotic stewardship. Drivers of over-the-counter antibiotic dispensing came from the demand side, such as customers sometimes asking for specific antibiotics and from the supply side where important influences included product promotion by pharmaceutical company representatives.

Most antibiotic stewardship programmes have centered around institutional settings where more control can be exerted [27]. However, in Bangladesh and other LMICs, institution prescriptions for antibiotics likely constitute a small proportion of those consumed [16–18]. Based on expert input from stakeholders, for an intervention to address antibiotic stewardship in Bangladesh, the two priority audiences were household members (customers) and drug shop staff; delivering an intervention at drug shops can reach both audiences. In Bangladesh and other LMICs, drug shops are the first point of healthcare contact [35].

A barrier to encourage community members to obtain a prescription is an inability to convey ‘registered physicians’, the term used in the National Drug Policy, 2016 [25] in simple terms, largely due to the community’s inability to distinguish between the various labels of ‘doctors’. There are many groups that are referred to as some type of doctor in the absence of formal qualification in both urban [11] and rural settings [12]. Despite this, community members likely understand that their local drug shop staff is not a fully qualified, practicing, medical practitioner that has completed medical school training. Moreover, there is confusion over training levels for people working in drug shops and almost no knowledge among consumers on policy. While access and cost remain important motivators to seek health care and relevant medications from the local drug shop, it seems impossible for drug shop staff and consumers to adhere to the current policy. Further advocacy among drug policy stakeholders to convey that several steps are needed to bridge the vast gulf between obtaining medical advice and antibiotics from drug shops (current practice) and obtaining prescriptions from a registered physician (policy targeted practice) is imperative for the country to move towards improved antibiotic stewardship.

We detected poor knowledge of relevant policies, including penalties for non-compliance, especially among drug shop staff, who are those predominantly affected. An international analysis found that poor governance and poor community infrastructure were both associated with higher AMR prevalence [36]. In Asia, rates of antibiotic dispensing without a prescription have changed little over the last 30 years [37], suggesting weak regulation. Thus, interventions that can improve policy knowledge and in turn can impact AMR should be considered along with lobby and advocacy efforts with relevant government agencies.

Policy on antibiotic prescribing needs to be considered along with the lack of an alternate route to medicines; restricting access to antibiotics purchased through unlicensed drug shops will have a negative effect on human health in communities where infectious disease rates remain high [17]. Thus, our expert group suggested that drug shop staff should be included when addressing antibiotic stewardship, even though government policy specifies that antibiotic prescriptions are acceptable from ‘registered physicians’ only. Access to registered physicians is not currently possible for large sectors of the population because the number of registered physicians is insufficient to meet demand [38, 39]. Cost and convenience are also important barriers for patients to seek care from qualified physicians [17, 30], echoed in the findings from piloting the SBCC intervention resources . These challenges are similar in other settings with over-the-counter provision of antibiotics [40, 41].

Drug shop staff have little incentive to comply with government policy. Drug shop staff need motivation to comply with licensing, the prescribing policy and minimum staff qualification, all of which are beyond the scope of an education based SBCC. The accredited drug dispensing outlet (ADDO) programme from Africa includes business incentives [42] which have the potential to offset perceived loss from compliance with a stewardship intervention.

Knowledge deficits were notable, and these are amendable to change using a SBCC intervention to improve health seeking behaviours, improve health literacy on antibiotic use and action and to inform drug shop staff about legal requirements. Training based programmes for drug shops have been conducted in Asia on dispensing medications for specific illnesses, with mixed results [37]. The ADDO programme that permits groups other than registered physicians to prescribe and dispense antibiotics may be a model that the Government of Bangladesh could consider. The cadre of government employed auxiliary healthcare providers (persons with 1–4 years of medical-related training) who are permitted to prescribe a limited number of drugs, some of which include antibiotics, could be further trained and mobilised to meet needs that fall between registered physicians and unqualified providers.

This study has several limitations. Respondent selection for data collection was based on convenience sampling and therefore was likely not representative of the population. Additionally, sites selected for formative data collection and SBCC pilots unlikely represented all geographic and socioeconomic populations. The data collection objective was to analyse as varied responses as possible. We ensured that data were collected from respondents from both urban and rural settings, where characteristics such as distance from healthcare facilities, population density and health seeking behaviours likely differed. A strength of the study was utilizing the behaviour change wheel as a systematic framework for data synthesis. In this study we describe data collection and synthesis to complete steps 1–4 outlined by Munir et al. [33] of the behaviour wheel [32] and the Com-B model for intervention development. The remaining aspects of step 5 and subsequent steps: identify behavioural change techniques; use APEASE criteria to grade these; identify mode of delivery, need to be explored in future research. Further refinement of intervention messages to improve antibiotic stewardship among drug shop staff and their customers could benefit from more interactive development such as using co-design methods. Drug shop staff will be unable to comply with an intervention that severely impacts profit and their livelihoods and further exploration of the economic aspects of an antibiotic stewardship intervention, relevant to this context, is necessary. This study focuses on intervention development for two of the four groups explored, based on priorities suggested by expert opinion. It may be necessary to involve the remaining groups that may either have greater affect or be easier to engage with on antibiotic stewardship in the future.

## Conclusions

This study drew on expert opinion and formative studies to guide development of an SBCC with priorities audiences; these need further collaboration with target audiences to refine messages and delivery methods. The study identified the likely poor penetration of relevant policy and penalties. Policies had limitations of flexibility to respond to the vast gap between current health seeking and prescribing practices at drug shops compared to recommendation of dispensing antibiotic only against prescription from registered physicians. Research that includes BPMI audit studies similar to those conducted in Africa [28] will further aid collaborative and advocacy efforts with relevant agencies in the Government of Bangladesh to improve antibiotic stewardship.

## Data Availability

The datasets used and/or analysed during the current study are available from the corresponding author on reasonable request.

## Abbreviations

ADDO: Accredited drug dispensing outlet
AMR: Antimicrobial resistance
MBBS: Bachelor of Medicine, Bachelor of Surgery
BPMI: Bangladesh Pharmacy Model Initiative
Com-B: Capability, Opportunities, Motivation-Behaviour
icddr,b: International Centre for Diarrhoeal Disease Research, Bangladesh
LMICs: Low- and middle-income countries
NGOs: Non-governmental organisations
SBCC: Social and behavioural change communication
VISCOM: Visual Communications Ltd
